# Insights on Ultrafiltration-Based Separation for the Purification and Quantification of Methotrexate in Nanocarriers

**DOI:** 10.3390/molecules25081879

**Published:** 2020-04-18

**Authors:** Sara S. Marques, Inês I. Ramos, Sara R. Fernandes, Luisa Barreiros, Sofia A. C. Lima, Salette Reis, M. Rosário M. Domingues, Marcela A. Segundo

**Affiliations:** 1 LAQV, REQUIMTE, Departamento de Ciências Químicas, Faculdade de Farmácia, Universidade do Porto, Rua de Jorge Viterbo Ferreira n 228, 4050-313 Porto, Portugal; micf09213@ff.up.pt (S.S.M.); iiramos@ff.up.pt (I.I.R.); saraferns@sapo.pt (S.R.F.); lbarreiros@ff.up.pt (L.B.); slima@ff.up.pt (S.A.C.L.); shreis@ff.up.pt (S.R.); 2 Escola Superior de Saúde, Instituto Politécnico do Porto, 4200-072 Porto, Portugal; 3 Centro de Espetrometria de Massa, Departamento de Química & QOPNA, Universidade de Aveiro, Campus Universitário de Santiago, 3810-193 Aveiro, Portugal; mrd@ua.pt; 4 Departamento de Química & CESAM & ECOMARE, Universidade de Aveiro, Campus Universitário de Santiago, 3810-193 Aveiro, Portugal

**Keywords:** drug delivery, encapsulation efficiency, permeation, separative methods, ultrafiltration

## Abstract

The evaluation of encapsulation efficiency is a regulatory requirement for the characterization of drug delivery systems. However, the difficulties in efficiently separating nanomedicines from the free drug may compromise the achievement of accurate determinations. Herein, ultrafiltration was exploited as a separative strategy towards the evaluation of methotrexate (MTX) encapsulation efficiency in nanostructured lipid carriers and polymeric nanoparticles. The effect of experimental conditions such as pH and the amount of surfactant present in the ultrafiltration media was addressed aiming at the selection of suitable conditions for the effective purification of nanocarriers. MTX-loaded nanoparticles were then submitted to ultrafiltration and the portions remaining in the upper compartment of the filtering device and in the ultrafiltrate were collected and analyzed by HPLC-UV using a reversed-phase (C18) monolithic column. A short centrifugation time (5 min) was suitable for establishing the amount of encapsulated MTX in nanostructured lipid carriers, based on the assumption that the free MTX concentration was the same in the upper compartment and in the ultrafiltrate. The defined conditions allowed the efficient separation of nanocarriers from the free drug, with recoveries of >85% even when nanoparticles were present in cell culture media and in pig skin surrogate from permeation assays.

## 1. Introduction

The promise held by nanomedicine of revolutionizing therapeutic outcomes has led to the extensive development of nanotechnology-based drug delivery systems [1,2,3]. These materials with dimensions in the nanometer range present unique properties and have been exploited as vehicles for the targeted delivery of pharmaceutical drugs, especially those with hampered therapeutic use [3,4,5]. Indeed, drug encapsulation circumvents solubility, degradation, and pharmacokinetic issues, increasing bioavailability at target tissues and decreasing toxicity caused by an off-target delivery. However, the translation of nanomedicines to the clinical context requires a precise characterization of drug delivery systems along with the reliable evaluation of nanoparticles behavior in biological media [3,5,6].

Due to the impact on quality and safety, the determination of the total amount of drug present in the final nanoformulation, along with the quantity associated with the nanoparticles (encapsulation efficiency), is a regulatory requirement [7,8]. However, an accurate encapsulation efficiency determination requires efficient separation of loaded nanoparticles from the free drug while maintaining nanoparticle properties, and this is still a challenging task [9,10]. Indeed, inefficient separations or precipitation/adsorption of the free drug during the separative process often lead to inaccurate results. Several methods have been employed for the separation of nanoparticles from the free drug, including size-exclusion chromatography, solid-phase extraction, X-ray small angle scattering (SAXS), ultrafiltration, ultracentrifugation, and dialysis [10,11,12,13,14]. The selection of the most suitable method should be carefully pondered considering nanoparticles and drug properties. In fact, drug leakage can occur during size-exclusion chromatography and during solid-phase extraction due to the extensive dilution and the interactions with the solid support [10]. Moreover, the long time required for equilibration between the compartments defined by the dialysis membrane [12,15], and the poor separation of nanoparticles from free drug even at a high centrifugation speed [9,12,15] often hamper the use of dialysis and ultracentrifugation as separation strategies. On the other hand, ultrafiltration does not require extensive centrifugation times or dilution of formulations [10,12], and it allows the separation of nanoparticles from free drug independently of nanoparticles density [9]. However, the use of suitable molecular weight cut-offs (that must be chosen from a limited range) and the absence of free drug interactions with nanoparticles and with filter materials must be ensured for efficient separations [16,17].

Additionally, online separation of nanoparticles from free drug with direct quantification of encapsulation efficiency has been pursued through hydrodynamic-based separations [11,18] and column-switching HPLC methods [15,19,20]. Nevertheless, those methods imply a priori characterization of the stability of encapsulated drugs during analysis to ensure that leakage does not occur. Moreover, adsorption of nanoparticles to a conventional stationary phase often precludes hydrodynamic separations [21,22].

Considering the features of currently available strategies for determination of encapsulation efficiency, it is consensual that there is no standard method that can be broadly applied for all types of nanoparticles. Moreover, the obtained results are dependent on assay conditions. Hence, method selection should be based on the properties of the nanocarrier and drug under study, with an extensive evaluation of experimental conditions to validate encapsulation efficiency results.

Methotrexate is a disease-modifying antirheumatic and chemotherapeutic drug, commonly used in the management of solid tumors, and autoimmune and inflammatory conditions [23,24,25]. It is a hydrophilic molecule [25] presenting low permeability and poor aqueous solubility, classified as a class IV drug according to the Biopharmaceutical System [24,26]. Despite being therapeutically effective, the use of methotrexate in the clinic is often limited due to its short half-life and associated toxic effects, which has motivated its vehiculation in drug-delivery systems, such as lipid and polymeric nanoparticles [23,25,27,28,29].

In this work, ultrafiltration was exploited towards the efficient separation of lipid and polymeric nanocarriers from the free methotrexate (MTX) regarding encapsulation efficiency determinations. This technique was selected due to its easy accessibility and promising separative efficiency under adequate experimental conditions. Therefore, a comprehensive study of the effect of the experimental parameters on the effective separation of free methotrexate from MTX-loaded nanostructured lipid carriers (NLCs) and poly(lactic-co-glycolic acid) (PLGA) nanoparticles was performed. Due to the challenges in the encapsulation of hydrophilic compounds, the development of efficient ultrafiltration procedures allowing effective purification of nanoparticles from the non-encapsulated drug will contribute to safer and better characterized nanomedicines.

## 2. Results and Discussion

### 2.1. Characterization of Nanoparticles and Total Methotrexate Content

#### 2.1.1. Size, Polydispersity, and Stability in Organic Solvent

NLCs and MTX-NLCs presented diameters of 208 ± 6 and 211 ± 9 nm, respectively, and monodisperse distributions (polydispersity index values < 0.1). Similarly, PLGA and MTX-PLGA nanoparticles presented diameters of 215 ± 5 and 218 ± 6 nm and polydispersity index values <0.02, also indicating a monodisperse distribution. The presence of 20% (*v*/*v*) acetonitrile (ACN, organic modifier in HPLC analysis) caused an increase in NLCs size to ca. 505 nm, with no decrease in the intensity of scattering signal measured by dynamic light scattering. This means that nanoparticles were still present, but their size/structure was changed. Higher amounts of acetonitrile (40% *v*/*v*) resulted in phase separation and in the decrease of scattering intensity to background levels, indicating NLCs disruption. Similarly, a decrease in the scattering intensity to background levels was verified when PLGA nanoparticles were analyzed in 20% (*v*/*v*) acetonitrile.

#### 2.1.2. Assessment of Total Methotrexate Content by HPLC

According to the US Food and Drug Administration, nanoparticle encapsulation efficiency consists of the amount of nanoparticle-associated drug in comparison to the total amount (free plus nanoparticle-associated drug) [9,10]. This is commonly determined assuming the quantity of drug added during nanoparticle preparation is the total amount. However, this assumption can be erroneous, especially when nanoparticles preparation involves several steps and part of the free drug is lost.

The total amount of MTX (free and nanoparticle-associated) present in the NLCs and PLGA nanoparticles under study was assessed. Nanoparticles were analyzed by HPLC after dilution in an ACN-phosphate buffer (pH 7.0; 0.1 M) (10:90, *v*/*v*) (Figures S1 and S2). The direct analysis of loaded hydrophobic nanocarriers by reversed-phase chromatography is based on the release of encapsulated compounds due to hydrophobic interactions between the nanocarriers and the hydrophobic stationary phase [15,20]. The analysis after nanoparticle disruption (acetonitrile at 40% and 20% (*v*/*v*) for MTX-NLCs and MTX-PLGA nanoparticles, respectively) provided similar results, indicating the validity of the HPLC method to determine total (free and nanoparticle-associated) MTX.

Hence, total MTX in the final emulsion of MTX-NLCs was 21.9 ± 0.7 µg, corresponding to 81% ± 2% of the theoretical value and indicating that about 20% of the drug is lost during the preparation steps. For MTX-PLGA nanoparticles, 16.2 ± 1.6 µg was found, corresponding to 93% ± 4% of the theoretical value and indicating a lower loss of the drug during nanoparticles preparation.

### 2.2. Establishment of Ultrafiltration Conditions

**Figure 1:**
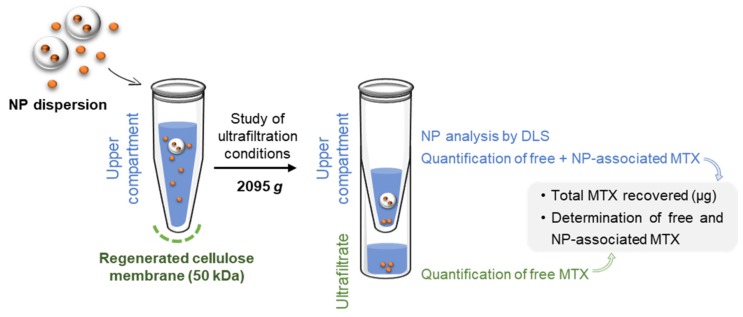
Experimental workflow for the quantification of total methotrexate (MTX) in nanoparticles (NP) dispersion, along with the separation and quantification of MTX-loaded in NPs and free MTX.

The assessment of nanoparticle encapsulation efficiency using ultrafiltration as a separative technique is based on the permeation of the free drug across the ultrafiltration membrane while nanoparticles are retained in the upper compartment (Figure 1). The separation depends on the selection of a filter pore size that enables only the permeation of the free drug, as well as on the establishment of ultrafiltration conditions that ensure the absence of interactions between free drug and filter components.

Therefore, as the selection of filter pore size relies on the size of nanoparticles, a 50 kDa pore size was used, enabling the passage of compounds up to 2.4 nm (free MTX < 1 nm) [30]. The possible interaction with filtering materials requires careful studies since it depends on the properties of the drug, properties of the filtering material, and also on the assay conditions. Indeed, drug-filter interactions could result in drug precipitation inside the filter pores, causing overestimated results when the encapsulation efficiency is determined solely based on the concentration of free drug in the permeate.

Likewise, if the drug retained in the filter is released when the upper compartment portion is recovered, the determined encapsulation efficiency values will also be overestimated. The global ultrafiltration process can be compromised if the precipitation or adhesion of compounds to the filter occur, decreasing the flow across the filter and making results time-dependent [10].

These issues require that studies consider not only the free amount of drug in the ultrafiltrate but also the determination in the upper compartment content, aiming to establish a mass balance for quality control.

#### 2.2.1. Effect of pH in the Ultrafiltration Process

First, the influence of pH on the permeation of free MTX through the filter was studied. MTX solutions were prepared in water (pH 5.5) and in phosphate buffer (pH 7.0) and submitted to ultrafiltration (5 min, 2095× *g*). A substantial loss of MTX (ca. 46%) was observed when ultrafiltration was performed in water (Table 1), with formation of a visible yellow precipitate in the filter. Higher recoveries were achieved when the buffer was used, indicating that pH affected the ultrafiltration process for this drug. In fact, 33% ± 1% of MTX was found in the upper compartment when ultrafiltration was performed in water whereas only 7.01% ± 0.01% remained when the phosphate buffer was used. Thus, ultrafiltration in water is not suitable for this drug. The change in the pH value does not affect the ionization of MTX molecule, with MTX existing in a doubly deprotonated form at both carboxylic groups (net charge −2) for pH 5.5 (97% of molecules) and 7.0 (100% of molecules) (Figure S3). Conversely, it has been described that the surface of cellulose membranes becomes more negatively charged when the pH of ultrafiltration media increases [31]. Although this effect of pH on regenerated cellulose zeta potential is not completely understood, the adsorption of anions seems to be a reasonable explanation. Hence, this would result in increased repulsive forces between the drug and the filter at pH 7.0, avoiding precipitation and accounting for the higher MTX recoveries at this pH.

Higher recoveries were achieved when similar tests were performed using NLC nanoparticles loaded with MTX (94% ± 13% in water and 96% ± 4% in a buffer), indicating no precipitation or interaction of MTX with the filter. Moreover, 73% and 59% of the total amount of drug present in the formulation were found in the upper compartment, indicating the stability of the lipid nanocarriers at both pHs for the timeframe of analysis. This observation is in agreement with other studies, where a similar MTX release pattern was found within 1 h of the assay for MTX-NLCs at pH 5.5 and 7.4, followed by an increased drug release for pH 5.5 in relation to 7.4 along the time, suggesting the higher stability of drug loaded content at pH 7.4 [27]. This is also in agreement with the higher stability of acidic drugs at pH 7.4 in relation to a more acidic environment [32].

Finally, the high recoveries attained (>94%) can be possibly attributed to the presence of a surfactant (polyvinyl alcohol) remaining from the procedure for preparation of nanoparticles, or these results can be due to a lower quantity of MTX available (because of its encapsulation) for filter interaction. Hence, ultrafiltration in a phosphate buffer was implemented for further assays.

#### 2.2.2. Effect of Polyvinyl Alcohol Content in the Ultrafiltration Process

The preparation of lipid and polymeric nanoparticles often comprises the use of a surfactant to stabilize the emulsion and to prevent nanoparticle agglomeration and precipitation [33,34,35,36]. Polyvinyl alcohol (PVA), a polymeric surfactant commonly used for this purpose, was employed during the preparation of the nanoparticles under study, at 6.7 mg mL^−1^ (NLCs) and 10.0 mg mL^−1^ (PLGA nanoparticles).

To study the effect of this surfactant on the ultrafiltration process, MTX solutions prepared in phosphate buffer containing 0.17, 0.75, and 2.5 mg mL^−1^ of PVA (corresponding to 0.34, 1.5, and 5.0 mg, respectively) were submitted to ultrafiltration for 5, 10, and 15 min. Recovery values for MTX were >98% for all conditions, considering the sum of MTX in the ultrafiltrate and in the upper compartment (Table S1). These recovery values were higher than the attained when the same analysis was performed without polyvinyl alcohol for the MTX solution (Table 1), also confirming that the surfactant decreased MTX-filter interactions.

**Figure 2:**
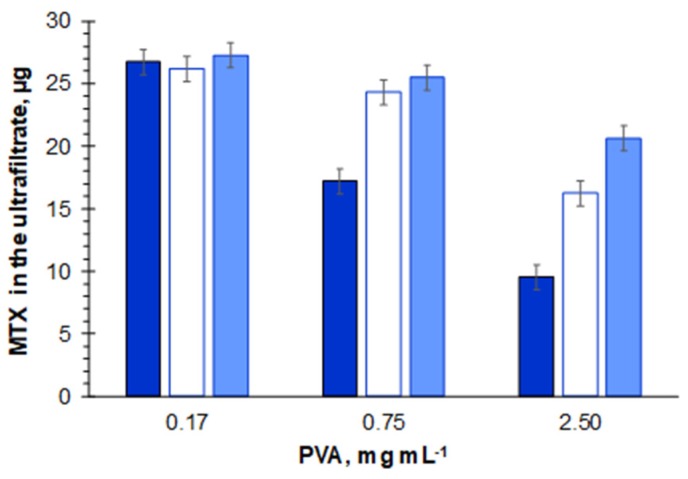
Mass (µg) of free MTX found in the ultrafiltrate after 5 (dark blue), 10 (white), and 15 (light blue) min of the ultrafiltration process when solutions of 0.17, 0.75, and 2.5 mg mL^−1^ of polyvinyl alcohol (PVA) containing free MTX (13.9 µg mL^−1^, corresponding to 27.8 µg) were submitted to ultrafiltration.

Despite the low interaction of the filter material, PVA markedly affected MTX permeation, with decreasing volumes (and consequent decreasing of MTX amounts) present in the ultrafiltrate after 5 min of centrifugation for increasing PVA quantities (Figure 2, Table S1). In fact, whereas for 0.17 mg mL^−1^ of PVA, 92% of the loaded volume permeated the filter after 5 min of centrifugation, for PVA concentrations of 0.75 and 2.5 mg mL^−1^, the permeation volume decreased to 62% and 35% of the initial volume, respectively. The increase in ultrafiltration time to 15 min was still insufficient for MTX ultrafiltration when 2.5 mg mL^−1^ of PVA were present (passage of 73% ± 3% of loaded volume).

This suggests that, at higher levels, polyvinyl alcohol hinders the ultrafiltration flow through the regenerated cellulose membrane with consequent accumulation of the loaded solution at the upper compartment of the filter. Previous studies regarding the ultrafiltration of polyvinyl alcohol (5–30 mg mL^−1^) through polysulfone membranes reported that the increase of PVA concentration resulted in decreased permeation flux due to the gel-layer formation [37]. Moreover, due to the broad molecular weight range of polyvinyl alcohol (30,000–70,000 Da), it is expected that PVA molecules above 50 kDa do not permeate the filter, remaining in the upper compartment.

Therefore, the amount of polyvinyl alcohol loaded onto filtering devices must be considered when ultrafiltration of nanoparticles is intended. A compromise between the minimum required amount of PVA to assure stabilization of particles and the maximum allowed for effective ultrafiltration should be pursued. Otherwise, dilution of nanoparticle dispersions can be performed to attain a polyvinyl alcohol amount tolerable by the ultrafiltration process. Considering the obtained results, a PVA concentration < 0.17 mg mL^−1^ is recommended for short (<5 min) centrifugation. Higher concentrations can be used, but the centrifugation time must also be increased.

#### 2.2.3. Effect of Nanoparticle Presence in the Ultrafiltration of Free Methotrexate

To study the effect of nanoparticles presence on the ultrafiltration of MTX, experiments were performed with blank nanoparticles spiked with MTX. Two levels of nanoparticles concentration were tested to elucidate about nanoparticles interference on MTX permeation through a filtering device.

**Figure 3:**
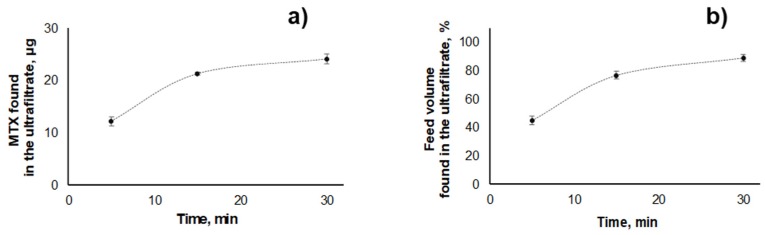
Effect of ultrafiltration time in the (**a**) MTX mass (µg), and (**b**) percentage of feed volume transferred to the ultrafiltrate compartment when blank NLCs (26 mg mL^−1^) spiked with 13.9 µg mL^−1^ MTX were submitted to ultrafiltration (2095× *g*).

For NLCs, total MTX recoveries of 99% ± 1% (27.7 ± 0.3 µg) were attained for the highest concentration (26 mg mL^−1^) of nanoparticles tested (Table S2). However, MTX distribution through the compartments was affected by the presence of these nanoparticles, with permeation of only 12.2 ± 0.9 µg of MTX (ca. 44% of total mass) after 5 min of centrifugation (ca. 33% less in relation to the assay performed using PVA). Additionally, only 45% ± 3% of the initial volume crossed the filter (Figure 3, Table S2). This suggests that the presence of NLCs hampers the flow through the ultrafiltration membrane. Indeed, an increased permeation was observed when the same amount of MTX was submitted to ultrafiltration in the presence of lower amounts of blank NLCs (five times less, 5.2 mg mL^−1^), with 73% ± 4% of total MTX and 81% ± 4% of the initial volume passing through the filter after 5 min of centrifugation (Table S2). In fact, pore constriction effects have been described before for regenerated cellulose modified by the inclusion of glyceryl triestearate [38] and lecithin-triestearine lipid nanoparticles [39]. Therefore, the effect of NLCs on ultrafiltration can be possibly attributed to lipid nanoparticle deposition among the chains of regenerated cellulose [40]. Hence, a longer centrifugation time (e.g., 30 min) is required to promote the passage of ≥85% of MTX mass and of initial volume through the filter when the highest tested concentration of NLCs (26 mg mL^−1^) is present in the ultrafiltration media (Figure 3).

To further explore this effect, blank NLCs spiked with MTX were submitted to ultrafiltration in an ACN-phosphate buffer (pH 7.0; 0.1 M) (10:90, *v*/*v*) mixture. The presence of acetonitrile in ultrafiltration media significantly changed the amount of MTX found in the ultrafiltrate in relation to a buffer (|t_calc_| = 18.60, t_tab_ = 2.78, ν = 3, *p* = 0.05). As the acetonitrile levels applied do not affect membrane flux resistance [41], this observed effect can be possibly attributed to ACN interaction with NLCs, hampering their deposition between regenerated cellulose chains and thus cake formation.

This delay on ultrafiltration was not observed for polymeric nanoparticles. In fact, 89% ± 7% of MTX mass and 93% ± 1% of initial volume passed through the filter after ultrafiltration during 15 min (Table S2) for these nanoparticles. Moreover, non-significant differences were found in the quantity of MTX passing through the filter when different concentrations of blank PLGA nanoparticles (15 and 76 mg mL^−1^) spiked with MTX were tested (|t_calc_| = 1.68, t_tab_ = 2.45, ν = 6, *p* = 0.05).

### 2.3. Determination of Nanoparticle-Associated Methotrexate

#### 2.3.1. Nanostructured Lipid Carriers (NLCs)

Since the presence of NLCs in the preparation media hampers the MTX passage through the filter, a centrifugation time of 30 min using the buffer or of 5 min for analysis in the ACN-phosphate buffer (pH 7.0; 0.1 M) (10:90, *v*/*v*) is required for the separation of free MTX from nanoparticles (>85% of MTX in ultrafiltrate). However, the effect of assay conditions on the encapsulated drug (e.g., occurrence of drug release from nanoparticles due to longer centrifugation and/or presence of organic solvent) may cause the underestimation of encapsulation values.

**Figure 4:**
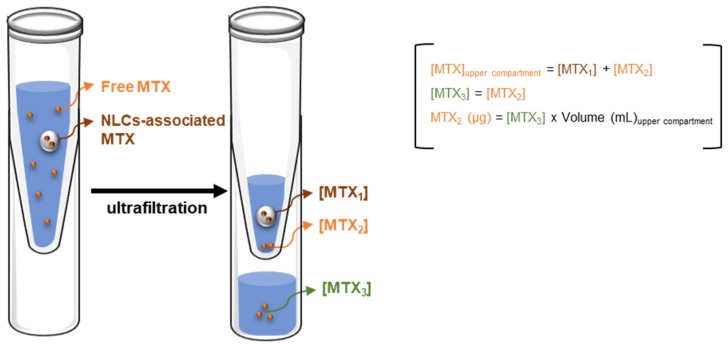
Schematic representation of the rationale applied for calculation of MTX-loaded in nanoparticles.

To address this issue, the quantity of MTX associated to nanoparticles was determined as the difference between the total and the free MTX found in the upper compartment (Figure 4, Table 2). Moreover, the determination of free MTX in the upper compartment was performed assuming that the concentration of free MTX in the upper compartment is equal to the concentration found in the ultrafiltrate (which contains only free MTX), and assuming also that no MTX-filter interactions occur (Figure 4).

These assumptions were further confirmed by the analysis of blank NLCs spiked with MTX submitted to ultrafiltration in buffer, with no significant differences (|tcalc| = 1.89, ttab = 2.45, ν = 6, *p* = 0.05) between MTX concentrations found in both compartments (14.0 ± 0.3 and 13.6 ± 0.3 µg mL^−1^ of MTX, upper compartment, and ultrafiltrate, respectively) after 5 min of centrifugation (Table S3).

Hence, quantification by HPLC of MTX in the content of upper compartment and in the ultrafiltrate was performed. As this strategy allows the determination of MTX associated to NLCs even when a complete separation from free MTX does not occur, the effect of ultrafiltration conditions (e.g., centrifugation time) on the stability of the encapsulated compound was studied, along with encapsulation efficiency determination after 5 min of centrifugation.

In fact, the increase in centrifugation time (from 5 to 30 min) in assays using the phosphate buffer caused a decrease in the quantity of MTX associated to NLCs from 0.96 ± 0.02 to 0.20 ± 0.09 µg (Table 2), suggesting MTX release from NLCs when longer centrifugation times are applied. Similarly, ultrafiltration in an ACN-phosphate buffer mixture for 5 min resulted in a decrease to 0.34 ± 0.01 µg of the MTX associated to NLCs. The integrity of NLCs was also checked by DLS, providing values of 222 ± 2 nm for the particles retained in the upper compartment and a background level signal for the ultrafiltrate, discarding the breakdown of nanoparticles into smaller structures.

The proposed strategy allowed the determination of MTX encapsulation efficiency in NLCs, with low levels found in phosphate buffer after 5 min of ultrafiltration (3.5 ± 0.1%). This low encapsulation value is in agreement with the low efficiency commonly attained for passive encapsulation of hydrophilic molecules, such as MTX (log P = −0.24) into hydrophobic carriers by single-emulsion procedures [33,34,42,43]. Thus, the preparation of these nanoparticles using double-emulsion procedures could improve the encapsulation values for this compound, as observed in other studies for MTX encapsulation in PLGA nanoparticles [23,25,29] and also for other hydrophilic compounds in lipid nanoparticles [44,45]. Likewise, other parameters such as the study of the effect of surfactant molecular weight, surfactant quantity, and pH of preparation media could also be exploited fostering better encapsulation values.

Nevertheless, it is important to consider that the low encapsulation values could also be a result of a burst release upon dilution and analysis, even for the short time of ultrafiltration used (5 min). In fact, in vitro release studies performed with MTX-NLCs with similar compositions and preparation procedures revealed a release of 64% [46] and 30% [27] of MTX in the first 2 h of dialysis for assays at pH 7.4 (37 °C). Moreover, a recent study described that a period of 4 h is required for the passage of free MTX through a dialysis bag membrane [47]. The potential of ultrafiltration to elucidate about the drug burst release from nanoparticles was previously recognized as an advantage in relation to dialysis due to the time required for the equilibration between the free drug and dialysis membrane [12]. Such an effect could justify the higher encapsulation efficiencies reported in the literature for MTX-NLCs, with encapsulations of 64% [46] and 87% [27] indirectly determined through quantification of the free drug, with release rates of 64% and 30% in 2 h, respectively.

#### 2.3.2. Polymeric Nanoparticles

The same methodology was applied for the determination of the MTX associated to PLGA nanoparticles, following the ultrafiltration conditions set for these nanocarriers. Therefore, PLGA nanoparticles (76 mg mL^−1^, 150 µL) were submitted to ultrafiltration in phosphate buffer for 15 min. Experiments with blank PLGA nanoparticles spiked with 8.95 µg mL^−1^ (corresponding to 17.9 µg) of MTX were also performed under the same conditions. No significant differences (|t_calc_| = 1.84, t_tab_ = 2.45, ν = 6, *p* = 0.05) were found between the concentration of MTX found in the upper compartment and in the ultrafiltrate when blank nanoparticles spiked with MTX were submitted to ultrafiltration (Table S4), proving the set strategy suitable for encapsulation efficiency determinations in PLGA nanocarriers.

However, the obtained results with the MTX-PLGA nanoparticles under analysis showed that encapsulation of MTX was <1% and could not be quantified by the proposed strategy. In fact, low encapsulation values (2%–3%) of MTX in PLGA nanocarriers have also been described elsewhere [29] using a more sensitive technique comprising nanoparticle disruption and extraction of MTX.

### 2.4. Determination of Nanoparticle-Associated Methotrexate in Complex Media

In order to evaluate the stability of encapsulated MTX when a nanoparticle analysis is performed in complex media from permeation assays, a batch of MTX-NLCs containing 2.0 ± 0.3 µg of MTX associated to NLCs (encapsulation efficiency of 7% ± 1%) was submitted to ultrafiltration for 5 min in pig skin surrogate media, DMEM, and DMEM-FBS cell culture media.

No matrix interferences were observed within the timeframe of MTX elution (Figure S4), with MTX recoveries ˃85%. No differences in the amount of MTX associated to nanoparticles were observed for assays in DMEM culture media in comparison to the buffer. However, when proteins were present the amount of MTX associated to nanoparticles decreased (Table 3). In fact, 1.3 ± 0.3 µg of MTX were associated to nanoparticles in a DMEM-FBS medium in comparison to 2.0 ± 0.3 µg determined when nanoparticles were present in the buffer. This decrease was more noticeable in pig skin surrogate (decrease ˃50%). This effect could have been caused by protein adsorption to the nanoparticles surface as suggested by the increase in nanoparticles size from 222 ± 5 to 289 ± 6 nm when assays were performed in pig skin surrogate.

## 3. Materials and Methods

### 3.1. Chemicals and Solutions

Dipotassium hydrogen phosphate, potassium dihydrogen phosphate, polyvinyl alcohol (PVA, MW = 30,000–70,000, 87%–90% hydrolyzed) and Dulbecco’s phosphate buffered saline (DPBS modified, without calcium chloride and magnesium chloride) were acquired from Sigma-Aldrich (St Louis, MO, USA). MTX was kindly supplied by Excella (Feucht, Germany). Witepsol^®^ E85 (mixture of hard fat compounds having a melting point above 37 °C) and Miglyol^®^ 812 (mixture of triglycerides, mainly caprylic acid and capric acid) used for NLCs preparation were purchased from Cremer Oleo (Hamburg, Germany). Poly(lactic-co-glycolic acid) (PLGA) (50:50 PURASORB^®^ PDLG 5004A) was kindly provided by Purac Biomaterials (Gorinchem, The Netherlands). Acetone (analytical grade) and acetonitrile (ACN, LiChrosolv HPLC grade) were purchased from VWR Chemicals (Radnor, PA, USA). Ultrapure water (resistivity > 18 MΩ cm) from the AriumPro system (Sartorius, Goettingen, Germany) was used in the preparation of all solutions. Amicon^®^ ultrafiltration devices (MW cut-off 50 kDa) were acquired from Merck Millipore (Merck, Darmstadt, Germany). Dulbecco’s modified Eagle medium (DMEM) and fetal bovine serum (FBS) were obtained from Gibco^®^ by Life Technologies™ (Invitrogen Corporation, Paisley, UK).

A phosphate buffer (pH 7.0; 0.1 M) composed of 0.2 M dipotassium hydrogen phosphate and 0.2 M of potassium dihydrogen phosphate was used for the preparation of the chromatographic mobile phase. MTX standards (0.05–25 µg mL^−1^) were prepared in an ACN-phosphate buffer (10:90, *v*/*v*). Similarly, NLCs and PLGA nanoparticles were analyzed after five times dilution in an ACN-phosphate buffer (10:90, *v*/*v*).

Pig skin surrogate media was prepared by placing porcine skin between the donor and acceptor compartments of a Franz diffusion cell (PermeGear, Hellertown, USA). Ten-times diluted Dulbecco’s PBS was added to donor (0.5 mL) and acceptor (5 mL) compartments, respectively. The porcine skin was maintained at 37 °C with stirring for 8 h to simulate the matrix of permeation assays. After the incubation period, the content of the acceptor compartment was collected and used for further assays.

### 3.2. Preparation and Characterization of Methotrexate-Loaded Nanocarriers

Methotrexate-loaded nanostructured lipid carriers (MTX-NLCs) were prepared by the hot ultra-sonication method as described before [27] with slight modifications. Briefly, 150 mg of Witepsol^®^ E85 (solid lipid) and 45 mg of Miglyol^®^ 812 (liquid lipid) were melted in a water bath at 65 °C. After lipid melting, 3.8 mg of MTX were added and the lipid phase was dispersed in 7 mL of a prewarmed PVA solution (6.7 mg mL^−1^). The resulting emulsion was submitted to probe-sonication (VCX130, Sonics and Materials, 115 Newtown, CT, USA) for 5 min (70% amplitude). The final nanoemulsion was cooled to room temperature. Blank NLCs were prepared similarly without MTX addition to the lipid phase.

Poly(lactic-co-glycolic acid) (PLGA) nanoparticles were synthesized using the single emulsion-solvent evaporation technique as described before [48]. Briefly, 20 mg of PLGA and 2.0 mg of MTX were dissolved in acetone (1.0 mL). The organic component was slowly added to 20 mL of 1% (*w*/*v*) PVA and submitted to probe-sonication for 1 min (70% amplitude). The resulting nanoemulsion was maintained under magnetic stirring at 300 rpm at room temperature overnight for acetone removal.

Nanoparticles were characterized regarding the hydrodynamic size and polydispersity by dynamic light scattering (DLS) using a ZetaPALS Particle Analyzer (Brookhaven Instrument Corps, Santa Barbara, CA). NLCs and PLGA nanoparticles were diluted in water (200 and 20 times, respectively) prior to a DLS analysis without any further treatment. The stability of nanoparticles when ACN was present in the media was assessed using ACN-phosphate buffer mixtures.

### 3.3. High-Performance Liquid Chromatography Method

The HPLC system and the chromatographic conditions used in this work were described in detail elsewhere [49]. The analysis of MTX standard solutions and diluted nanoparticle batches (analyzed without any pretreatment or after ultrafiltration process) were performed in a Jasco HPLC system (Easton, USA) composed by a PU-2089 pump, an AS-2057 autosampler, a LC-Net II/ADC controller, and a Jasco MD-2015 photo diode array detector. The chromatographic analysis was performed using a reversed-phase monolithic column (Chromolith^®^ RP-18e, 100 × 4.6 mm id, Merck, Darmstadt, Germany) preceded by a guard column of the same material (5 × 4.6 mm id). The separation was accomplished in 3.5 min using an ACN-phosphate buffer (pH 7.0; 0.1 M) (9:91, *v*/*v*) as the mobile phase. Determinations were performed using an injection volume of 50 μL, a flow rate of 1.5 mL min^−1^, and 302 nm as detection wavelength. Additionally, nanoparticles were also analyzed by gradient elution to evaluate if other chromatographic peaks were detected upon increasing the ACN content (leading to nanoparticles disruption) [21]. For that purpose, mixtures of 0.5 M of phosphate buffer (mobile phase A), ACN (mobile phase B), and ultrapure water (mobile phase C) were performed online, ensuring 20% (*v*/*v*) of mobile phase A during the whole chromatographic run (constant buffer content). The ACN content was maintained during the first 5 min (9% (*v*/*v*)), followed by an increase from 5–25 min up to 50% (*v*/*v*), which was held until 35 min of the run, after which the initial conditions were restored.

### 3.4. Determination of Methotrexate in Nanoformulations

The total, free, and nanoparticle-associated MTX present in the nanoemulsions under study were determined as depicted in Figure 1. The total content of MTX (comprising free MTX and nanoparticle-associated MTX) in the nanoformulations was assessed by HPLC. For that purpose, ca. 52 and 152 mg of NLCs and PLGA nanoparticles, respectively, were sampled and diluted in 2 mL of ACN-phosphate buffer (pH 7.0; 0.1 M) (10:90, *v*/*v*). Expected theoretical values, corresponding to 100% MTX present in the nanocarrier, were calculated based on the mass of nanoformulation used for HPLC analysis and the mass percentage (% *w*/*w*) of MTX used for the preparation of nanoformulations.

Ultrafiltration was used to differentiate free and nanoparticle-associated MTX. Amicon^®^ ultrafiltration devices (MW cut-off 50 kDa) composed by regenerated cellulose membranes were used. All devices were washed with water before use as recommended by manufacturers to remove glycerine residues. NLCs and PLGA nanoparticle batches were diluted to a final volume of 2 mL (NP dispersion) and immediately submitted to centrifugation at 2095× g (Allegra^®^ X-15R Centrifuge, Beckman Coulter, Brea, CA, USA). Solutions of MTX with different amounts of surfactant (polyvinyl alcohol) and dispersions of blank nanoparticles spiked with free MTX were also submitted to ultrafiltration for comparison purposes.

After centrifugation, the portion remaining in the upper compartment of the filter and the liquid present in the ultrafiltrate compartment (portion that passed through the filtering device) were collected. The mass and volume of each fraction were measured for further calculations. Both fractions were analyzed by HPLC for the determination of MTX (µg) distribution through filter compartments. The fraction collected from the upper compartment after ultrafiltration of nanoparticles was also analyzed by dynamic light scattering to inspect size alterations.

Concerning the application to samples, 50 µL (corresponding to 52 mg) of NLCs and 150 µL (corresponding to 152 mg) of PLGA nanoparticle dispersions were diluted in pig skin surrogate media (final volume of 2 mL) and submitted to ultrafiltration. The MTX recovery, volume, and mass present in each compartment of the filter were determined. For assays in the DMEM-FBS media, dispersions containing 130 mg mL^−1^ of NLCs and PLGA nanoparticles were prepared in DMEM-FBS, followed by dilution in a phosphate buffer (5 times) prior to ultrafiltration.

## 4. Conclusions

In this work, the total quantity of MTX present in lipid and polymeric nanoparticles was assessed using reversed-phase chromatography. Further quantification of free and nanoparticle-associated MTX was performed through the establishment of suitable ultrafiltration conditions for the separation of lipid and polymeric nanoparticles from free MTX. Indeed, when ultrafiltration was performed in buffered media (pH 7.0), the interaction between free MTX and filtering device was decreased compared with the experiments at a lower pH (5.5) and no buffering. Moreover, the time required for free MTX permeation was dependent on the amount of surfactant present in the nanoformulation. Regardless, due to their composition, lipid nanoparticles hampered the ultrafiltration process, requiring a longer centrifugation time than polymeric nanoparticles for separation from free MTX.

The determination of nanoparticle-associated MTX using ultrafiltration as a separative technique under the set assay conditions revealed low encapsulation levels. This may be explained by non-optimal nanoparticle preparation procedures or a drug burst release. The second is often masked when dialysis is used as a separative technique due to the time required for membrane equilibration. The set ultrafiltration conditions were suitable for the separation of lipid nanoparticles from free MTX in complex media (pig skin surrogate, DMEM, and DMEM-FBS culture media), proving the method suitable for application to samples from permeation assays, including acceptor media where contributions from the free and encapsulated drug may be distinguished.

Therefore, this work provides valuable insights for the implementation of effective ultrafiltration procedures towards the efficient separation of nanoparticles from free drugs. Due to the easy accessibility of ultrafiltration membranes and experimental simplicity, ultrafiltration is a suitable separative method towards the determination of nanoparticle encapsulation efficiency and release values, provided that adequate experimental conditions are applied. Advances in this field will contribute to improved nanoparticle characterization, a demand for successful clinical translation.

## Figures and Tables

**Table 1:** Assessment of MTX present in the upper compartment and ultrafiltrate for MTX solutions ^1^ and methotrexate-loaded nanostructured lipid carriers (MTX-NLCs ^2^) submitted to ultrafiltration ^3^ in different media.

	MTX Solution		MTX-NLCs	
	Water	Buffer ^4^	Water	Buffer ^4^
MTX upper compartment (µg)	5.0 ± 0.5	1.5 ± 0.1	14 ± 1	12.5 ± 0.1
MTX ultrafiltrate (µg)	10 ± 1	20.3 ± 0.1	4.2 ± 0.3	8.1 ± 0.9
Total MTX (µg)	15 ± 1	21.9 ± 0.2	19 ± 1	21 ± 1
MTX recovery (%)	54 ± 5	79 ± 1	94 ± 13	96 ± 4
Vol. upper compartment (mL)	0.10 ± 0.01	0.10 ± 0.01	1.0 ± 0.1	1.1 ± 0.1
Vol. ultrafiltrate (mL)	1.9 ± 0.1	1.9 ± 0.1	1.0 ± 0.1	0.8 ± 0.1

^1^ MTX concentration of 13.9 µg mL^−1^, corresponding to 27.8 µg; ^2^ HPLC determined values of MTX for NLCs-MTX analyzed were 20 ± 2 µg (water) and 21.3 ± 0.4 µg (buffer); ^3^ 5 min, 2095× *g*; ^4^ potassium phosphate buffer (pH 7.0; 0.1 M).

**Table 2:** Determination of NLC-associated MTX when dispersions of MTX-NLCs ^1^ were submitted to ultrafiltration in buffer and acetonitrile-buffer media.

Ultrafiltration Media	Time (min)	Total MTX Upper Compartment (µg)	Free MTX Upper Compartment (µg)	MTX Associated to NLCs (µg)	Encapsulation Efficiency (%)
Buffer ^2^	5	12.46 ± 0.09	11.50 ± 0.07	0.96 ± 0.02	3.5 ± 0.1
Buffer ^2^	30	3.48 ± 0.01	3.28 ± 0.09	0.20 ± 0.09	0.7 ± 0.3
10% (*v*/*v*) acetonitrile	5	4.6 ± 0.1	4.22 ± 0.01	0.34 ± 0.01	1.24 ± 0.04

^1^ Dispersions (2 mL) containing 50 µL of MTX-NLCs (corresponding to 26 mg mL^−^^1^); ^2^ potassium phosphate buffer (pH 7.0; 0.1 M).

**Table 3:** Determination of NLC-associated MTX in cell culture media and pig skin surrogate.

	Total MTX Upper Compartment (µg)	Free MTX Upper Compartment (µg)	MTX Associated to NLCs (µg)
Buffer ^1^	12.5 ± 0.1	10.6 ± 0.3	2.0 ± 0.3
Pig Skin Surrogate	14.4 ± 0.4	13.64 ± 0.03	0.8 ± 0.4
DMEM	13.8 ± 0.2	11.6 ± 0.1	2.2 ± 0.2
DMEM-FBS	12.1 ± 0.3	10.81 ± 0.01	1.3 ± 0.3

^1^ Values obtained in buffer for this batch of MTX-NLCs are presented for comparison.

## Supplementary Materials

The following are available online. Figure S1: Analysis of a) NLCs and b) PLGA nanoparticles. Chromatograms from i) mobile phase, ii) 0.5 µg mL^−1^ MTX solution, iii) blank nanoparticles, and iv) MTX-loaded nanoparticles are depicted, Figure S2: Chromatograms obtained from the analysis of a) mobile phase, b) blank NLCs, and c) MTX-NLCs using the gradient elution. Mobile phase A, phosphate buffer (pH 7.0, 0.5 M); mobile phase B, acetonitrile; mobile phase C, ultrapure water. Gradient: 20% A during all the chromatographic run, 9% B from 0–5 min, increase until 50% B from 5–25 min, and return to the initial conditions from 35–50 min, Figure S3: Effect of pH in the charge and ionization of methotrexate. Data obtained through the chemicalize platform (https://chemicalize.com), Figure S4: Chromatograms from the analysis of a) mobile phase, and MTX-NLCs remaining in the upper compartment after ultrafiltration in b) potassium phosphate (pH 7.0, 0.1 M), c) pig skin surrogate, d) DMEM culture media, and e) DMEM-FBS culture media, Table S1: Effect of polyvinyl alcohol (PVA) in the ultrafiltration of MTX solutions, Table S2: Permeation of free MTX in spiked formulations, Table S3: Total and free MTX (µg) present in the upper compartment when MTX-NLCs and blank NLCs spiked with MTX were submitted to ultrafiltration, Table S4: Total and free MTX (µg) present in the upper compartment when solutions of MTX-PLGA and blank PLGA nanoparticles spiked with MTX were submitted to ultrafiltration.
