# Supplementary material for: Insights on Ultrafiltration-Based Separation for the Purification and Quantification of Methotrexate in Nanocarriers
Source: Molecules. 2020 Apr 18;25(8):1879. doi: 10.3390/molecules25081879 (PMC7221554; doi:10.3390/molecules25081879)
Supplement: Supplementary file 1 [file molecules-25-01879-s001.pdf]

Supplementary Information

Insights on ultrafiltration based-separation for the purification and quantification of methotrexate in nanocarriers

Sara S. Marques[1], Inês I. Ramos[1], Sara R. Fernandes[1,2], Luisa Barreiros[1,2], Sofia A.C. Lima[1], Salette Reis[1], M. Rosário M. Domingues[3,4], Marcela A. Segundo[1*]

[1]LAQV, REQUIMTE, Departamento de Ciências Químicas, Faculdade de Farmácia, Universidade do Porto, Porto, Portugal

[2]Escola Superior de Saúde, Instituto Politécnico do Porto, Porto, Portugal

[3]Centro de Espetrometria de Massa, Departamento de Química & QOPNA, Universidade de Aveiro, Campus Universitário de Santiago, 3810-193 Aveiro, Portugal

[4]Departamento de Química & CESAM & ECOMARE, Universidade de Aveiro, Campus Universitário de Santiago, 3810-193 Aveiro, Portugal

*Correspondence should be addressed to msegundo@ff.up.pt. Tel: +351 220428676 Fax: +351 226093483.

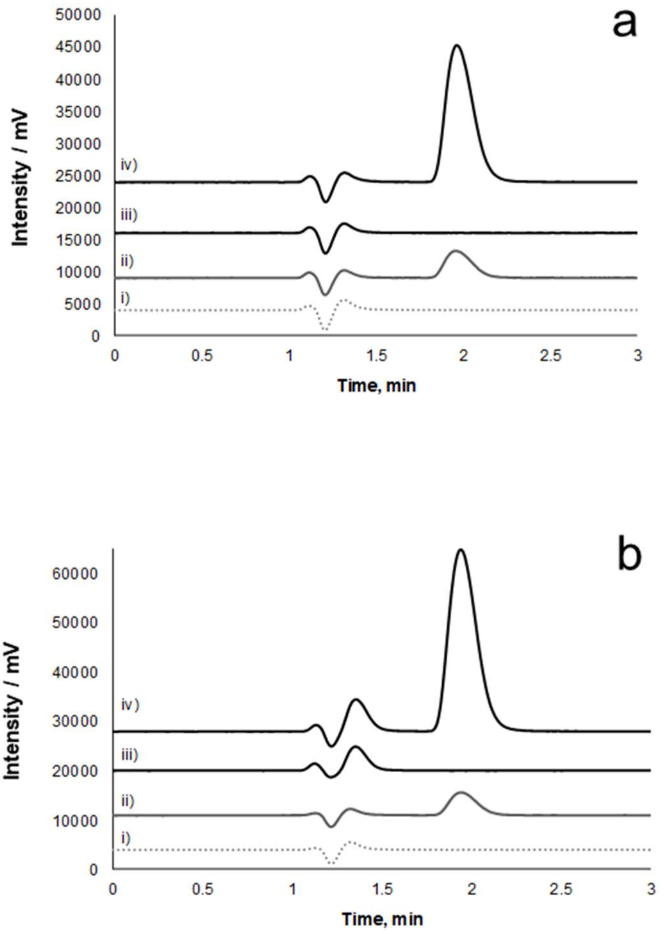

Fig. S1. Analysis of a) NLCs and b) PLGA nanoparticles. Chromatograms from i) mobile phase, ii) 0.5 µg mL^{-1} MTX solution, iii) blank nanoparticles, and iv) MTX-loaded nanoparticles are depicted.

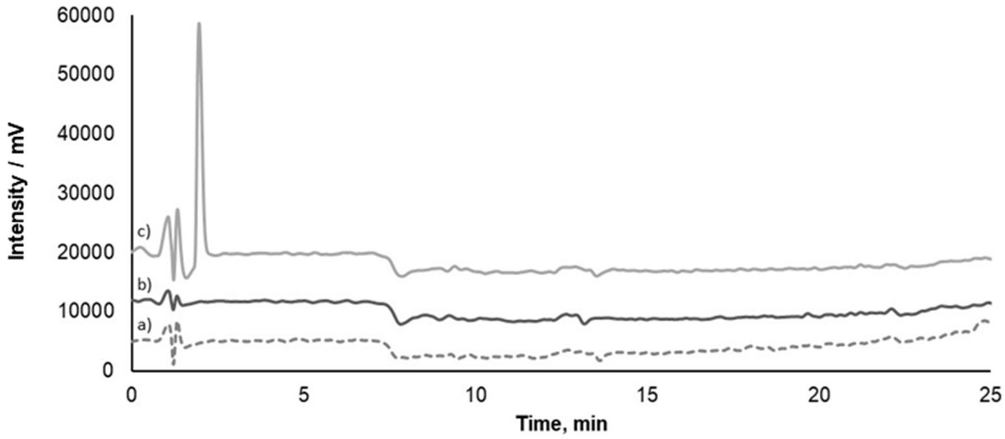

Fig. S2. Chromatograms obtained from the analysis of a) mobile phase, b) blank NLCs, and c) MTX-NLCs using gradient elution. Mobile phase A, phosphate buffer (pH 7.0, 0.5 M); mobile phase B, acetonitrile; mobile phase C, ultrapure water. Gradient: 20% A during all the chromatographic run, 9% B from 0-5 min, increase until 50% B from 5-25 min and return to the initial conditions from 35-50 min.

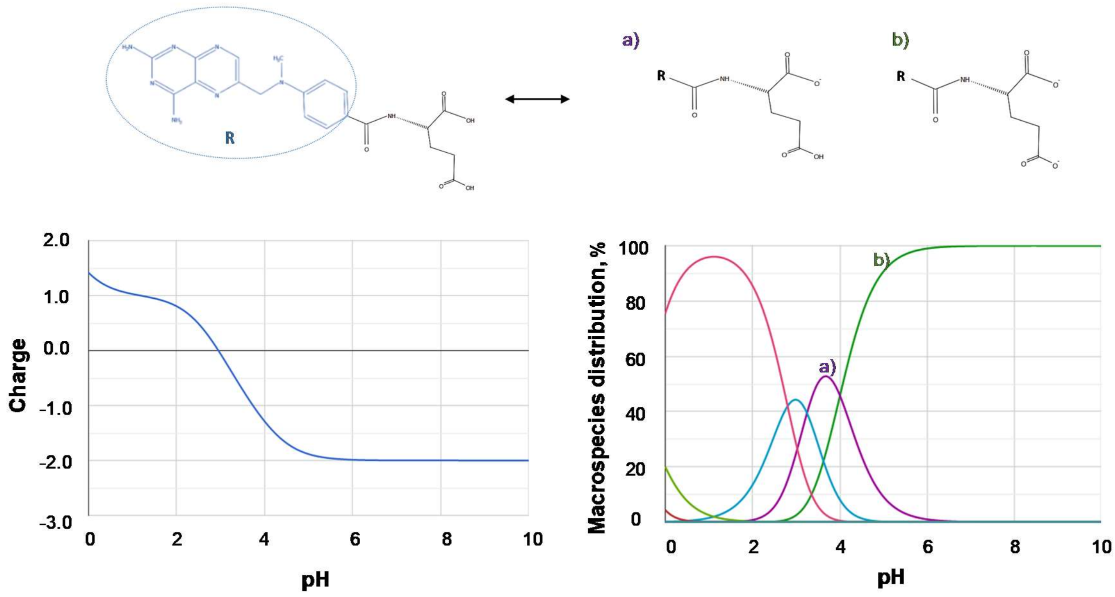

Fig. S3. Effect of pH in the charge and ionization of methotrexate. Data obtained through chemicalize platform (https://chemicalize.com).

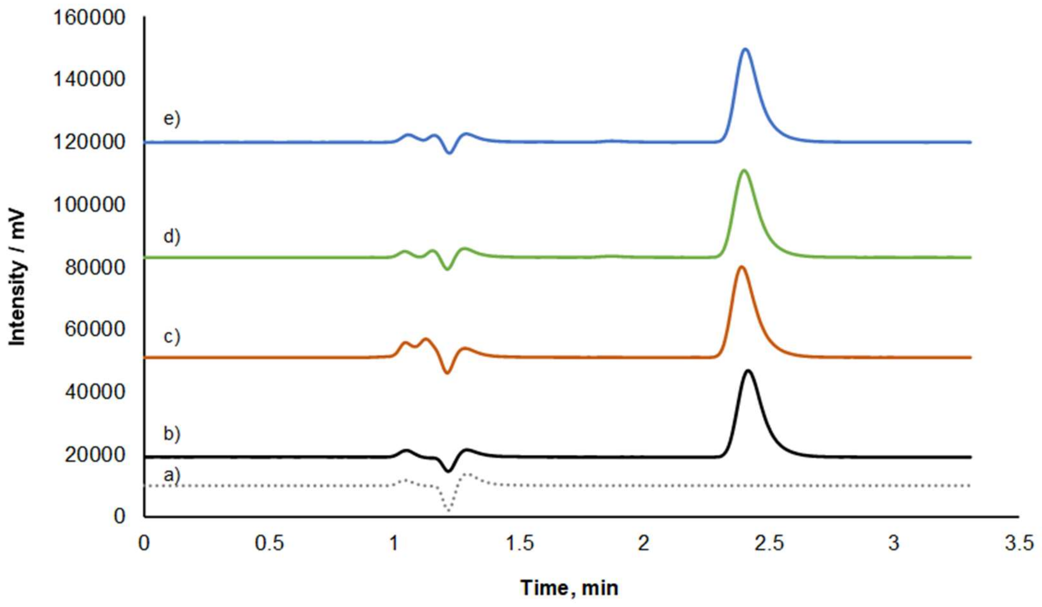

Fig. S4. Chromatograms from the analysis of a) mobile phase, and MTX-NLCs remaining in the upper compartment after ultrafiltration in b) potassium phosphate (pH 7.0, 0.1 M), c) pig skin surrogate, d) DMEM culture media and e) DMEM-FBS culture media.

Table S1. Effect of polyvinyl alcohol (PVA) in the ultrafiltration of MTX solutions.

**Ultrafiltration time (min)**		**5**			**10**			**15**	
**Polyvinyl alcohol concentration (mg mL^{-1})** [a]	**0.17**	**0.75**	**2.5**	**0.17**	**0.75**	**2.5**	**0.17**	**0.75**	**2.5**
MTX upper compartment (µg)	3.0 ± 0.5	11 ± 1	18.2 ± 0.8	0.59 ± 0.01	3.4 ± 0.4	12 ± 1	0.45 ± 0.02	2.0 ± 0.2	8 ± 1
MTX ultrafiltrate (µg)	27 ± 1	17.2 ± 0.1	9.5 ± 0.5	26 ± 1	24.3 ± 0.2	16 ± 1	27 ± 1	25 ± 1	21 ± 1
Volume recovered in the ultrafiltrate (%)	92 ± 4	62 ± 3	35 ± 4	98 ± 1	85 ± 1	58 ± 2	98 ± 1	93 ± 1	73 ± 3
Total MTX (µg) [b]	28 ± 1	28.3 ± 0.5	27.7 ± 0.2	27 ± 1	28 ± 1	28.1 ± 0.2	28.5 ± 0.2	29 ± 1	28.3 ± 0.3
MTX recovery (%) [c]	101 ± 4	102 ± 2	100 ± 1	98 ± 3	101 ± 3	101 ± 1	103 ± 1	103 ± 2	102 ± 1

[a] corresponding to 0.34, 1.5 and 5.0 mg of PVA, respectively.

[b] calculated as MTX upper compartment + MTX ultrafiltrate.

[c] value based in total MTX compared to the theoretical value of 27.8 µg of MTX.

Table S2. Permeation of free MTX in spiked formulations.

	NLCs[a]		PLGA[b]	
	low level[c]	high level[c]	low level[d]	high level[d]
MTX in the ultrafiltrate (µg)	20 ± 1	12.2 ± 0.9	17 ± 1	16 ± 1
Feed MTX permeation (%)	73 ± 4	44 ± 3	96 ± 5	89 ± 7
Feed volume permeation (%)	81 ± 4	45 ± 3	97.9 ± 0.3	93 ± 1
MTX recovered (µg)	26.2 ± 0.1	27.7 ± 0.3	18 ± 1	17 ± 1
MTX recovery (%)	94 ± 1	99 ± 1	99 ± 7	96 ± 7

[a] Blank NLCs spiked with 13.9 µg mL^{-1} of MTX (corresponding to 27.8 µg) and submitted to ultrafiltration (5 min, 2095 × *g*) in potassium phosphate (pH 7.0, 0.1M).

[b] Blank PLGA nanoparticles spiked with 8.95 µg mL^{-1} of MTX (corresponding to 17.9 µg) and submitted to ultrafiltration (15 min, 2095 × g) in potassium phosphate (pH 7.0; 0.1M).

[c] Low and high levels correspond to 5.2 and 26 mg mL^{-1} of NLCs, respectively.

[d] Low and high levels correspond to 15.2 and 76 mg mL^{-1} of PLGA nanoparticles, respectively.

Table S3. Total and free MTX (µg) present in the upper compartment when MTX-NLCs and blank NLCs [a] spiked with MTX[b] were submitted to ultrafiltration.

Ultrafiltration condition	Sample	Volume ultrafiltrate (mL)	Volume upper compartment (mL)	Total MTX upper compartment (µg)	Free MTX upper compartment (µg)
**Buffer[c], 5 min**	MTX-NLCs	0.80 ± 0.05	1.14 ± 0.04	12.5 ± 0.1	11.5 ± 0.1
	Blank NLCs spiked	0.90 ± 0.04	1.1 ± 0.1	15.6 ± 0.5	15.1 ± 0.4
**Buffer[c], 30 min**	MTX-NLCs	1.79 ± 0.03	0.24 ± 0.07	3 ± 1	2.4 ± 0.9
	Blank NLCs spiked	1.9 ± 0.2	0.3 ± 0.1	3 ± 1	3 ± 1

[a] Solutions containing 26 mg mL^{-1} of NLCs (50 µL of NLCs dispersed in 2 mL of the ultrafiltration media under analysis).

[b] 13.9 µg mL^{-1}

[c] Potassium phosphate (pH 7.0, 0.1 M)

Table S4. Total and free MTX (µg) present in the upper compartment when solutions of MTX-PLGA and blank PLGA nanoparticles[a] spiked with MTX[b] were submitted to ultrafiltration[c].

	Volume ultrafiltrate (mL)	Volume upper compartment (mL)	Total MTX upper compartment (µg)	Free MTX upper compartment (µg)
MTX-PLGA NPs	1.84 ± 0.04	0.16 ± 0.02	1.4 ± 0.2	1.3 ± 0.2
Blank PLGA spiked	1.87 ± 0.01	0.15 ± 0.02	1.3 ± 0.1	1.2 ± 0.1

[a] Solutions containing 76 mg mL^{-1} of PLGA nanoparticles (150 µL of PLGA nanoparticles dispersed in 2 mL of buffer).

[b] 8.75 µg mL^{-1}

[c] Potassium phosphate (pH 7.0, 0.1 M), 15 min, 2095 × *g*.
